# Multifocal gastric adenocarcinoma in a patient with LRBA deficiency

**DOI:** 10.1186/s13023-017-0682-5

**Published:** 2017-07-18

**Authors:** Nina Bratanič, Jernej Kovač, Katka Pohar, Katarina Trebušak Podkrajšek, Alojz Ihan, Tadej Battelino, Magdalena Avbelj Stefanija

**Affiliations:** 1 Department of Pediatric Endocrinology, Diabetes and Metabolism, University Medical Centre, University Children’s Hospital, Bohoriceva 20, 1000 Ljubljana, Slovenia; 2 University Medical Centre, University Children’s Hospital, Unit for Special Laboratory Diagnostics, Vrazov trg 1, 1000 Ljubljana, Slovenia; 3 University of Ljubljana, Faculty of Medicine, Institute for Microbiology and Immunology, Zaloška 4, 1000 Ljubljana, Slovenia; 4 University of Ljubljana, Faculty of Medicine, Vrazov trg 2, 1000 Ljubljana, Slovenia

**Keywords:** Lipopolysaccharide-responsive, Beige-like anchor (LRBA), Immunodeficiency, Autoimmunity, Gastric cancer, Malignant melanoma, Cytotoxic T-lymphocyte associated protein 4 (CTLA4), Malignancy

## Abstract

**Background:**

Lipopolysaccharide-responsive, beige-like anchor protein (LRBA) deficiency is characterized by primary immunodeficiency and autoimmunity. Cancer may present another feature of LRBA deficiency. We describe a case history of a young adult with LRBA deficiency and two independent malignancies.

**Methods:**

Family-trio whole exome sequencing with unbiased phenotype ontology approach was used for identification of causative mutations of a primary immune deficiency disorder. Additionally, we sought to identify germline mutations in genes known to be associated with two independent malignancies using a targeted approach. A cytotoxic T-lymphocyte associated protein 4 (CTLA4) expression in T lymphocytes was determined by flow cytometry.

**Results:**

In the patient with clinical signs of LRBA deficiency multifocal gastric carcinoma and malignant melanoma were diagnosed and surgically treated at 19 and 27 years of age, respectively. Despite refusal of any adjuvant chemotherapy or radiotherapy, the patient demonstrated disease free survival for at least 13 years after the first cancer diagnosis. A homozygous frameshift deletion in *LRBA* gene (p.Glu946Ter) and two common variants in *TYR* gene were identified. Reduced CTLA4 expression in a subset of regulatory T lymphocytes was identified in the patient and his unaffected mother carrying a heterozygous *LRBA* mutation as compared to control in a dose-dependent manner.

**Conclusion:**

This is the first description of gastric cancer and malignant melanoma in a young adult with LRBA deficiency. The role of *LRBA* gene knockout in cancer development and its prognosis remains to be elucidated.

**Electronic supplementary material:**

The online version of this article (doi:10.1186/s13023-017-0682-5) contains supplementary material, which is available to authorized users.

## Background

Deleterious germline mutations in *LRBA* gene encoding lipopolysaccharide-responsive, beige-like anchor protein (LRBA) have been recently associated with an autosomal recessive monogenic disorder, whose common denominators are **L**RBA deficiency, **A**utoimmunity, regulatory **T** (Treg) cell defects, **A**utoimmune **I**nfiltration, and **E**nteropathy (LATAIE syndrome) [1–5]. To our knowledge, more than 60 patients with LRBA deficiency have been reported, with a plethora of diverse mutations identified and with highly variable clinical and immunologic characteristics [1–18]. Several neoplasms are reported in LRBA deficiency: Burkitt lymphoma [10], low-grade *Ebstein Barr virus* positive (EBV+) marginal zone lymphoma [17], lymphomatous central nervous system pseudotumor [2], dysplastic tubular adenoma and polyps [18], and immunoproliferative diseases [4, 7, 8], suggesting that proliferative diseases may present another feature of LRBA deficiency.

We describe clinical, immunologic, and genetic characteristics of a patient with a novel pathogenic homozygous *LRBA* gene mutation presenting not only with immune deficiency and multiorgan autoimmunity, but also with two independent malignant diseases. The presented clinical spectrum recapitulates and extends the previously described phenotypes.

## Methods

### Patient

The clinical history of a 32-year old Caucasian male patient, followed up since infancy, is described. Clinical data were obtained from the medical records upon prior patient’s written consent. Genetic analysis of his pedigree was performed after the patient and his parents gave written informed consent approved by the Republic of Slovenia National Medical Ethics Committee.

### Genetic analysis

Whole exome sequencing (WES) was performed in Eurofins Genomics (Ebersberg, Germany) using Ion AmpliSeq Exome kit for whole exome enrichment preparation and Ion PI™ Sequencing 200 Kit v3 together with Ion Proton Sequencer (Thermo Fisher Scientific, Waltham, MA, USA).

Genetic variants with coverage >15× were analyzed with Variant Studio 2.2 software (Illumina, San Diego, CA, USA). A combination of family trio approach and phenotype driven analysis with Human Phenotype Ontology database [19] was used to direct and focus the analysis on *LRBA* genetic variants as shown in Additional file 1: Figure S1. The minor allele frequency threshold for known variants was set at 1%, and all variants exceeding this value were excluded from further analysis. Family segregation analysis with De novo and Autosomal Recessive inheritance model was used to further reduce the number of potential causative variants. Identified candidate variant and its family segregation was confirmed by a targeted Sanger sequencing run on ABI Genetic Analyzer 3500 (Applied Biosystems, Waltham, MA, USA) using custom oligonucleotides and BigDye Terminator v3.1 sequencing kit (Applied Biosystems, Waltham, MA, USA). In addition, genes associated with inherited susceptibility to malignant melanoma (*CDKN2A*, *MDM2*, *CDK4*, *RB1*, *MC1R*, *TYR*, *TYRP1* and *ASIP*) [20], gastric cancer (*CDH1*, *CTNNA1*, *BRCA1*, *BRCA2*, *APC*, *TP53*, *STK11*, *SMAD4*, *BMPR1A*, *MSH2*, *MLH1*) [21], and *CTLA4* gene were analyzed with targeted approach.

The Loss of Heterozygosity (LoH) was analyzed to evaluate the potential degree of consanguinity. Using HomSI algorithm [22], the WES data were analyzed for region specific increased ratio of homozygous variants.

### Cytotoxic T-lymphocyte associated protein 4 (CTLA4) expression by flow cytometry

Total and mobilized CTLA4 levels were determined as described by Lo et al. [10]. Briefly, peripheral blood mononuclear cells (PBMC) from the patient, carrier and healthy donor were isolated from whole blood using Ficoll-Paque gradient separation (GE Healthcare, Sweden). The collected cells were resuspended at 1 × 106 cells/mL in cell culture media (RPMI, 5% FCS, 1% penicillin/streptomycin, 1% L-glutamine). Total CTLA4 levels were determined by fixing and permeabilizing (BioLegend FoxP3 staining kit) freshly isolated cells, staining for CTLA4 and FoxP3 and surface markers, including cluster of differentiation (CD) 3, CD4 and CD25 and analyzed by flow cytometry. Mobilized CTLA4 levels were determined by stimulating the cells in the presence of anti-CTLA4 antibody (BD Biosciences, San Jose, California, USA) with 20 ng/mL phorbol 12-myristate 13-acetate (PMA) (Sigma-Aldrich, St. Louis, Missouri, USA) and 1 μM ionomycin (Sigma-Aldrich, St. Louis, Missouri, USA) for 30 min at 37 °C and 5% carbon dioxide (CO_2_) in the medium. Cells were then washed, fixed, permeabilized and stained as described above.

## Results

### Case report

**Fig. 1:**
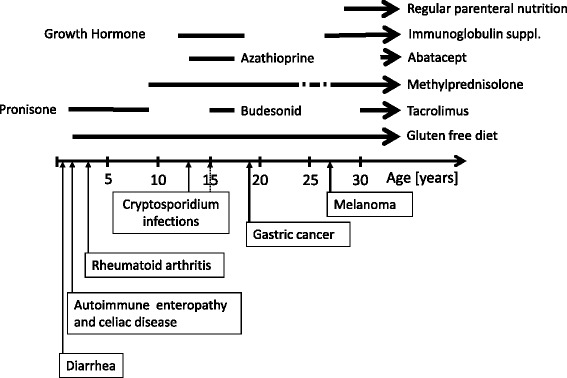
Schematic representation of the course of selected treatments and age at occurrence of selected disease manifestations. Arrows indicate ongoing therapy. Broken lines indicate two periods of pulse therapy with methyprednisolone

Propositus was born as the only child in the family in which no cancer or immune deficiency in the family history was reported. Parents are distant relatives; the patient’s great grandmother and great grandfather were first cousins. In support to their distant relation larger stretches of LoH regions were identified at chromosomes 2, 5 and 6 (Additional file 2: Figure S2). The patient presented at the age of 3 months with severe protracted diarrhea, hypoproteinemia and anemia. At the age of 18 months, total villous atrophy was found by endoscopic biopsy, but the result of serologic test for celiac disease was negative. A gluten free diet was introduced without significant clinical improvement. In addition to autoimmune enteropathy, the patient developed anti-nuclear antibodies (ANA) positive oligoarticular rheumatoid arthritis at the age of 3 years. Corticosteroid treatment was started which resulted also in a clinical improvement of intestinal manifestations. At the age of 10 years, autoimmune enteropathy was confirmed by gastrointestinal histology of jejunum, where mild villous atrophy and moderate crypt hyperplasia were seen. By immunohistochemistry equal number of plasma cells, immunoglobulin (Ig) A and IgM positive cells and the presence of IgE positive cells in lamina propria were determined; furthermore granular immune complexes along epithelial basal membrane were identified. Another immunohistochemic evaluation of colon biopsy performed at the age of 23 years demonstrated infiltration of mucosa with CD8+ cells and loss of enteroendocrine cells, but eosinophilic infiltrates or CD20+ B cells were not present. Since 3 years of age any attempt to taper steroids led to recurrent chronic diarrhea, so the patient was continuously treated with therapeutic doses of steroids, mostly without a protection with proton pump inhibitors until a diagnose of gastric cancer. Furthermore, malabsorption progressed and short bowel syndrome developed. At the age of 28 years, when his enteral losses increased to 3000 ml per day and he started to lose his weight, the patient could be persuaded to start treatment with parenteral nutrition in addition to enteral feeding. Various attempts of systemic or topical immunosuppression with azathioprine and budesonide to treat his enteropathy were of limited and/or short-term success. After the introduction of treatment with tacrolimus, the quantity of enteral losses diminished from 3000 - 4000 ml (125–160 ml/kg body weight/day) to 1500 ml per day (62,5 ml/kg body weight/day), which, after the introduction of abatacept, further diminished to 600 ml per day (25 ml/kg body weight/day). Schematic representation of selected therapy and major disease manifestations is shown in Fig. 1.

In addition to autoimmune enteropathy and rheumatoid arthritis, the patient had megaloblastic anemia with vitamin B12 deficiency responsive to monthly intramuscular vitamin B12 injections, primary hypothyroidism and atopic dermatitis. The results of autoantibodies evaluated to diagnose autoimmunity are briefly summarized in Additional file 3: Table S1, but anti-intrinsic autoantibodies were never determined. The patient had extreme growth retardation with final height of 123 cm with normal growth hormone secretion and was unresponsive to growth hormone therapy. He also had failure to thrive, Cushingoid face, osteoporosis, very thin skin with subcutaneous calcinations, nephrocalcinosis, cholelithiasis, arterial hypertension and a cataract, likely as complications of a long term corticosteroid treatment (and/or chronic disease). The patient was treated with high doses of growth hormone for 7 years and, despite increasing the dose of growth hormone, insulin-like growth factor 1 (IGF-1) and insulin-like growth factor binding globuline 3 (IGFBP3) levels reached only the average age and sex adjusted levels.

During the follow-up, we observed gradual decline in the number of B cells and immunoglobulin levels**.** Low B and T lymphocytes subpopulations and hypogammaglobulinaemia confirmed immunodeficiency, with low serum IgG and IgM levels and normal IgA. After 26 years of age monthly intravenous replacement of immunoglobulins was required. As shown by flow cytometry almost complete absence of B cells, markedly reduced number of T cells, CD4+ and CD8+ T cell subsets and Natural killer (NK) cells were demonstrated before the patient’s first cancer diagnoses (Additional file 4: Table S2). The number of naive Th (CD45 RA) and CD25 + CD4+ T cells was significantly reduced. T cell proliferative responses to phytohemagglutinin (PHA) were reduced, but after stimulation with CD3/CD28 normal T cell proliferative response was observed. Extensive immunophenotyping in recent years demonstrated that 96% of T lymphocytes HLA-DR were activated, suggestive of a hyperactive immune state.

The patient suffered from several opportunistic infections, including *Campylobacter jejuni, Morganella morganii, Proteus mirabilis, Yersinia enterocolitica*, *Cryptospordium parvus, Giardia lamblia, Legionella pneumophila*, pulmonary aspergillosis and aspergilloma, and chronic mucocutaneous candidiasis. He had immunologically confirmed *Ebstein Barr virus* primo infection at the age of 21 years. In addition, he had recurrent episodes of catheter sepsis caused by *Staphyloccocus epidermidis*.

At the age of 19 years, an abdominal ultrasound revealed a gastric tumor arising from the anterior and posterior wall of the gastric corpus that almost obstructed the outflow (Additional file 5: Figure S3). Subtotal gastrectomy with Roux-en-Y gastrojejunostomy with extended lymph node dissection was performed (1/36 positive). Histopathological findings (Additional file 5: Figure S3) confirmed multifocal invasive gastric adenocarcinoma, arising in a background of two separate adenomas with a high grade dysplasia. Carcinoma was mostly of an intestinal type (Lauren) and of an infiltrative type (Ming). Muscularis propria infiltration (pT2a) and lymphangiosis carcinomatosa were observed. At least two additional focuses of intramucosal carcinoma were identified in the surrounding mucosa. The dysplasia was present in a background of a chronic, diffuse active gastritis with intestinal metaplasia. Immunohistochemical studies for *Helicobacter pylori* were negative. Further treatment with chemo- and radiotherapy was suggested, which the patient declined. By the time of a publication 13 years later during regular oncological and radiological follow-up, no signs of progression of gastric carcinoma were observed. At the age of 27 years, the patient had excision of a 4 mm wide malignant melanoma in situ, located at the right ankle. The patient had fair skin and fair hair. No sunburns were reported in his history as well as very little sunlight exposure.

### Genetic analysis

Using whole exome sequencing with phenotype ontology approach a homozygous small frameshift deletion in *LRBA* (NM_006726.4: c.2836_2839delGAAA; NP006717.2: p.Glu946Ter, rs533294277, minor allele frequency in ExAC database [23] 1,66 × 10^−5^) was identified in the patient, confirmed by Sanger sequencing. The mutation introduces a premature stop-codon and is therefore considered pathological. Both parents were heterozygous carriers. The average coverage in region of interest captured by WES was 58.4X, 57.2X, 53.9X for ﻿the﻿ patient, his mother and father, respectively.

Additionally, compound heterozygosity for p.Ser192Tyr (NM_000372.4: c.575C > A, rs1042602) and p.Arg402Gln (c.1205G > A, rs1126809) polymorphisms in the tyrosinase gene (*TYR*) (minor allele frequency 0,12 and 0,08, respectively, according to dbSNP database, [24]) was determined, p.Ser192Tyr being inherited from the father and p.Arg402Gln from the mother. No other potentially pathogenic variants were identified in other melanoma or gastric cancer predisposing genes or in *CTLA4* gene.

### T cell subtype specific and LRBA dose-dependent reduction of CTLA4

**Fig. 2:**
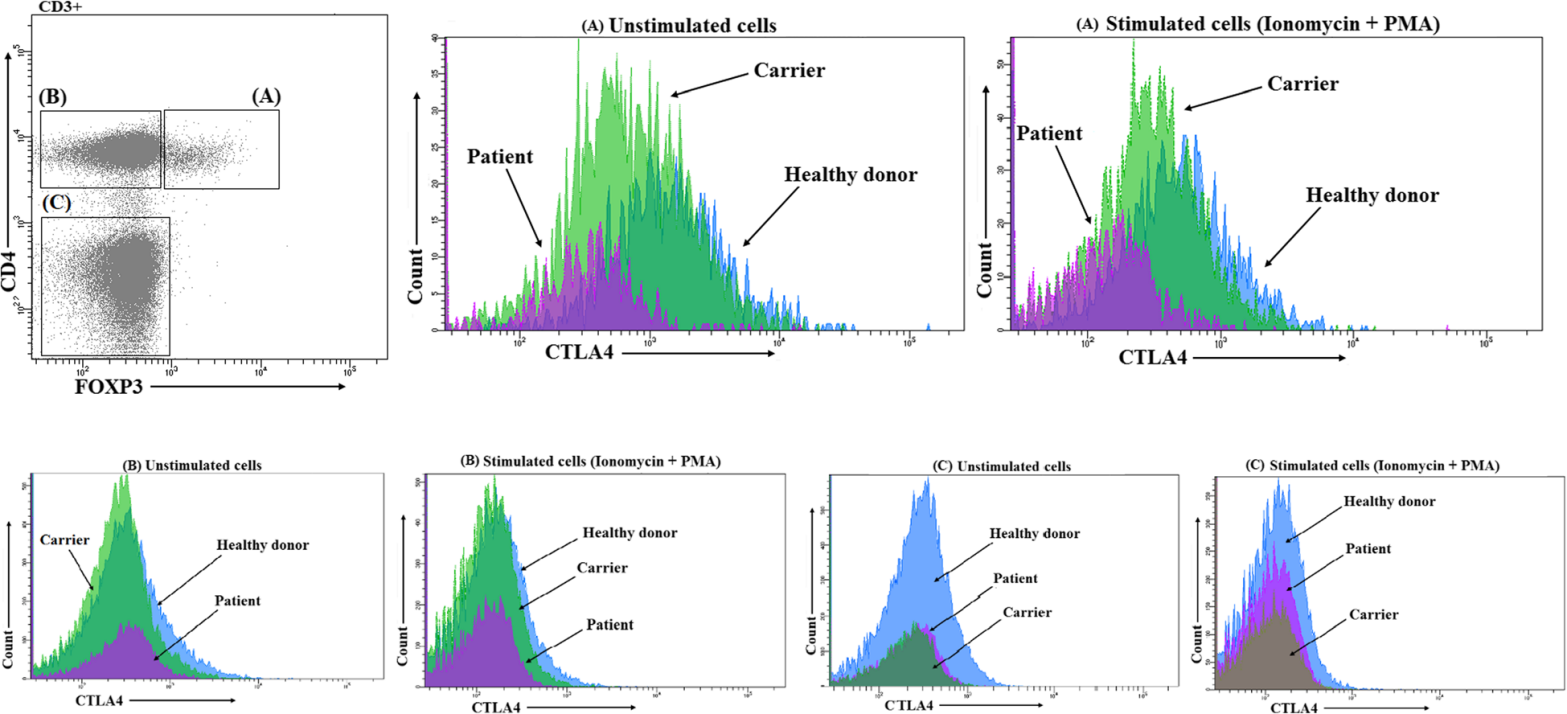
Total and mobilized CTLA4 levels in CD3+ cells. Unstimulated and stimulated FOXP3+ CD4+ T cells (A), FoxP3 negative CD4 T cells (B), and CD4 negative T cells (C) from LRBA deficient patient (*violet*), heterozygous carrier (*yellow*) and healthy donor (*blue*). Legend: PMA - phorbol 12-myristate 13-acetate

FoxP3+ cells were assessed on freshly isolated (unstimulated) and stimulated PBMC (stimulation with ionomycin and PMA) by flow cytometry, as described [10]. A markedly reduced CTLA4 expression on Treg cells (CD3+ CD4+ FoxP3+) was demonstrated in the patient, compared to healthy donor (Fig. 2). Furthermore, a reduction of CTLA4 expression in Treg cells was identified also in a heterozygous clinically unaffected mother. The CTLA4 mean fluorescence intensity (MFI) on FOXP3 + CD4+ T cells was highest in the healthy donor, lower in the heterozygous carrier and lowest in the LRBA deficient patient (Table 1). Interestingly, a reduction of CTLA4 expression in the patient and carrier was identified only in FoxP3+ T cells (Treg), while FoxP3 negative CD4 T cells (non – regulatory T helper cells) and CD4 negative T cells (T cytotoxic cells) expressed lower level of CTLA4 expression, and also no difference in the patient, carrier and healthy donor was measured (Table 1).

**Table 1:** The quantification of total and mobilized CTLA4

Status		Mean Fluorescence Intensity (MFI) of CTLA4 on FOXP3+ CD4+ T cells	MFI of CTLA4 on FOXP3- CD4+ T cells	MFI of CTLA4 on CD3 + CD8+ T cells
HEALTHY DONOR	UNSTIMULATED	2340	490	315
	IONO + PMA	695	178	119
CARRIER	UNSTIMULATED	1152	324	214
	IONO + PMA	393	121	89
PATIENT	UNSTIMULATED	501	331	215
	IONO + PMA	176	106	85

*MFI* mean fluorescence intensity, *IONO* ionomycin, *PMA* phorbol 12-myristate 13-acetate

At the time of CTLA4 expression analysis, the patient was more than six months off abatacept therapy.

## Discussion

Biallelic mutations in *LRBA* were previously implicated as a cause of common variable immunodeficiency with autoimmunity in humans. Like in many other primary immunodeficiency disorders, there is no clear genotype-phenotype correlation in patients with *LRBA* mutations. Multifocal gastric cancer and malignant melanoma were so far not reported in patients with *LRBA* gene mutations.

Our patient had several risk factors for developing gastric carcinoma. First, he had a “common variable immunodeficiency (CVID)-like condition” with multiple chronic infections and inflammation. The emergence of malignancies in primary immunodeficiency disorders results from the interplay between the underlying genetic defect, immune dysregulation with defective immunosurveillance mechanisms, and increased susceptibility to specific viruses [25]. Specifically, patients with CVIDs have a 10-fold increased risk of gastric cancer. The mechanisms are not fully understood, but the increased risk has been linked to a number of CVID-associated factors, including pernicious anemia, gastric atrophy, achlorhydria, decreased gastric IgA, and chronic Helicobacter pylori infection [26, 27]. The average age of onset of gastric carcinoma in patients with CVID is earlier than in those without immunodeficiency (46 years vs. 69 years) [28], but still much later then in our patient. Second, prolonged therapy with corticosteroids likely worsened existing immune dysfunction and increased the risk for cancer. Third, our patient had a recurrent infection with *Cryptosporidium*. The study on dexamethasone treated severe combined immunodeficiency (SCID) mice demonstrated that parasite *C. parvum* induced digestive adenocarcinoma [29].

The association of elevated growth hormone levels with gastric cancer has not been observed in either growth hormone treated patients or in patients exposed to extreme endogenous growth hormone secretion due to acromegaly [30].

Multiple cancers at a relatively young age might be associated with inherited cancer predisposition. Particularly, gastric cancer at such an early age is rare and likely attributed to increased sensitivity. Malignant melanoma on the other hand is a common cancer in Slovenian population with a crude incidence rate of 24,1/100.000 in a 5-year interval from 2009 to 2013. Furthermore, it is the most common cancer in Slovenian population younger than 30 years with 4,2% of all newly diagnosed melanoma patients being younger than 30 years [31]. The patient is a carrier of two common variants in *TYR* gene, p.Ser192Tyr and p.Arg402Gln of which only the variant p.Arg402Gln was associated with increased risk for melanoma in one large population study with odds ratio (OR) 1,21 [32], but not in two other similar studies [33, 34]. On the other hand, many primary immunodeficiency disorders are associated with elevated risks for different types of cancer [26]. Therefore, association of malignant diseases in our patient with LRBA deficiency can be suspected.

LRBA plays a major immunoregulatory role by helping maintain intracellular stores of CTLA4, which allows protein to mobilize rapidly to the cell surface [10]. CTLA4 is a critical and potent inhibitor of T-cell proliferation that serves as a “checkpoint” of immune responses. ***C*** *TLA4* gene **h**aploinsufficiency with **a**utoimmune **i**nfiltration (CHAI) leads to broad clinical manifestations such as hypogammaglobulinemia, enteropathy, recurrent infections, lymphocytic infiltration, and multiple autoimmune clinical features, very similar to characteristics of LRBA deficiency [5, 35–37]. Interestingly, 3 of the 24 (12.5%) cases with *CTLA4* haploinsufficiency reported developed gastric cancer and 2 of 3 patients presented with multifocal adenocarcinomas associated with atrophic gastritis and intestinal metaplasia, similarly to our patient [35–37]. We did not identify any potentially pathogenic variants in propositus’s *CTLA4* gene, but a markedly reduced CTLA4 expression by Treg cells was demonstrated by flow cytometry, as demonstrated previously in LRBA deficient patients [10]. Furthermore, a reduction of CTLA4 expression in Treg cells was identified also in a heterozygous clinically unaffected mother, which is a demonstration of an unopposed gene dosage effect of LRBA gene mutations in vivo. Even so Treg CTLA4 expression reduced by half seems to be clinically silent. Interestingly, *CTLA4* gene haploinsufficiency is disease-causing, while *LRBA* gene haploinsufficiency state in propositus’s mother caused no apparent disease until her current age of 54 years. Of note, no reduction in CTLA4 expression in the patient as compared to the control was demonstrated in FoxP3 negative CD4 T cells (non – regulatory T helper cells) and CD4 negative T cells (cytotoxic T cells). This suggests LRBA has a T cell subtype specific influence on CTLA4 expression, which may have importance in the disease mechanism. Both cell populations expressed lower level of CTLA4 expression. Differential CTLA4 expression in human CD4+ versus CD8+ T cells was described recently demonstrating significantly higher CTLA4 expression in the CD4+ T cells than in CD8+ T cells [38]. CTLA4 is higher at the protein and the transcriptional level in CD4+ T cells. These findings demonstrate a differential regulation of CTLA4 on CD4+ and CD8+ T cell subsets, which is likely important to the clinical efficacy for anti-CTLA4 therapies.

Intriguingly, LRBA deficiency and *CTLA4* haploinsufficiency could represent human in vivo models of CTLA4 blockade with checkpoint inhibitors; medicines that actually improved survival in several cancers, particularly in melanoma, but also in gastric cancer [39, 40]. CTLA4 serves as a critical checkpoint inhibitor as it downregulates T cell activation to prevent autoimmunity and allow tolerance to self-antigens. Wang JW et al. showed that LRBA is significantly upregulated in multiple tumor types [41]. Its expression and function are important for cancer cell proliferation and apoptosis. Inhibition of LRBA function may repress cancer cell growth in many cancer cell lines, but not all. These findings suggest that LRBA knockdown, when combined with cancer therapeutics, may achieve greater therapeutic effect than either entity alone. According to Wang JW et al. [41] we would expect that these patients are protected against cancer. Of note, our patient declined chemotherapy, yet he survived more than 13 years without reappearance of the disease, which is significantly longer survival than expected according to reports. Little is known concerning the long-term survival of adolescents and young adults with solid gastric cancers. Survival of 21 patients (12 females, 9 males) between 5 and 21 years of age ranged from 1 to 25 months [42]. In general, survival of immunodeficiency-associated gastric cancer in adult populations is longer, but still rarely over 10 years [28].

## Conclusions

In conclusion, to the best of our knowledge, we present the first co-occurrence of gastric cancer and malignant melanoma with LRBA deficiency. Despite the risk factors, including multifocal gastric cancer with muscularis propria invasion, lymphangiosis carcinomatosa, a positive lymph node, young age of onset, additional cancer and immunodeficiency, our patient demonstrated long cancer-free survival with only surgical treatment. The possible role of *LRBA* gene knockout in cancer development and its prognosis remains to be elucidated. The identification of key molecules that regulate cellular immune processes may enable development of novel immunotherapeutic approaches to cancer treatment.

## Additional files

Additional file 1: Figure S1. Workflow of WES filtering process with multiple filtering steps including a phenotype driven filtering. (PDF 308 kb)

Additional file 2: Figure S2. The identified regions of homozygous stretches in chromosome 2, 5 and 6. Description: Blue regions indicate homozygous variants and yellow regions indicate heterozygous variants. Orange regions in parental chromosomes (F, M) indicate heterozygous variants corresponding with homozygous variants of sibling’s genotype (P). (TIFF 898 kb)

Additional file 3: Table S1. Autoantibodies evaluated in the patient. (DOCX 12 kb)

Additional file 4: Table S2. Selected immune system determinants longitudinally evaluated in the patient. (DOCX 15 kb)

Additional file 5: Figure S3. Ultrasound and histopathologic images of the gastric cancer. Description: **(a.)** Ultrasound image of a 49 × 44 mm large tumor formation in the stomach; **(b., c.)** histopathologic images of bioptic material from the gastric tumor demonstrating intestinal type gastric carcinoma with lamina propria invasion (Kreyberg trichrom stain, 20X magnification (b.) 100X magnification (c.)). The square in part b. indicates the area of magnification in part c. of the figure. (PDF 223 kb)

## Abbreviations

ANA: Anti-nuclear autoantibodies
CD: Cluster of differentiation
CHAI: *CTLA4* gene Haploinsufficiency with Autoimmune Infiltration
CTLA4: Cytotoxic T-lymphocyte associated protein 4
CO2: Carbon dioxide
CTLA4: Cytotoxic T-lymphocyte associated protein 4
CVID: Common variable immunodeficiency
DNA: Deoxyribonucleic acid
EBV: *Ebstein Barr virus*
EDTA: Ethylenediaminetetraacetic acid
Ig: Immunoglobulin
IGF-1: Insulin-like growth factor 1
IGFBP3: Insulin-like growth factor binding globulin 3
LATAIE: Autoimmunity, regulatory T (Treg) cell defects, Autoimmune Infiltration, and Enteropathy
LoH: Loss of Heterozygosity
LRBA: Lipopolysaccharide-responsive, beige-like anchor protein
MFI: Mean fluorescence intensity
NK: Natural killer cell
OR: Odds ratio
PBMC: Peripheral blood mononuclear cells
PHA: Phytohemagglutinin
PMA: Phorbol 12-myristate 13-acetate
SCID: Severe combined immunodeficiency
WES: Whole exome sequencing
