# Supplementary figures and images for: Multifocal gastric adenocarcinoma in a patient with LRBA deficiency
Source: Orphanet J Rare Dis. 2017 Jul 18;12:131. doi: 10.1186/s13023-017-0682-5 (PMC5516372; doi:10.1186/s13023-017-0682-5)

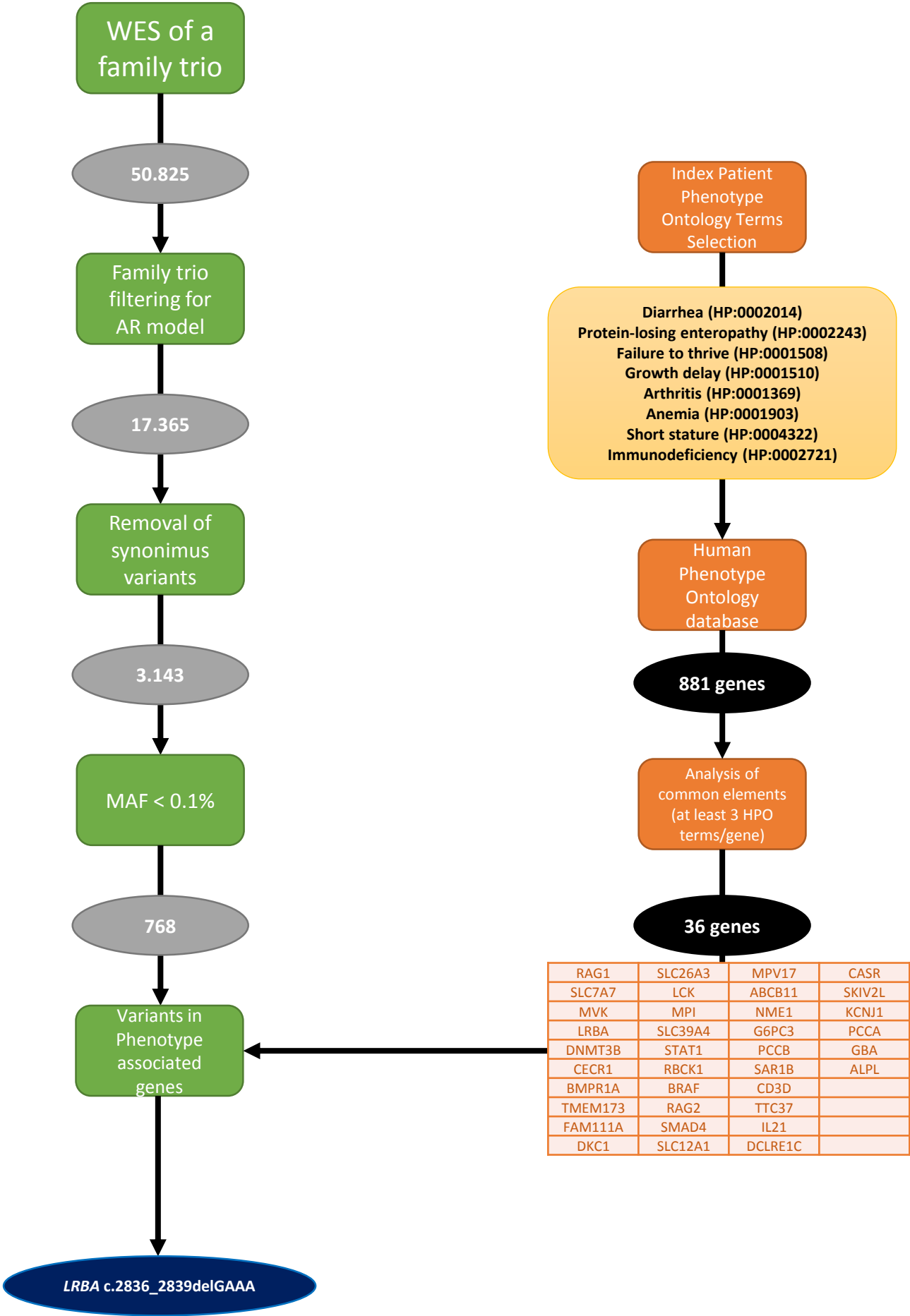

Supplement: Supplementary file 1 — Workflow of WES filtering process with multiple filtering steps including a phenotype driven filtering. (PDF 308 kb) [file 13023_2017_682_MOESM1_ESM.pdf]

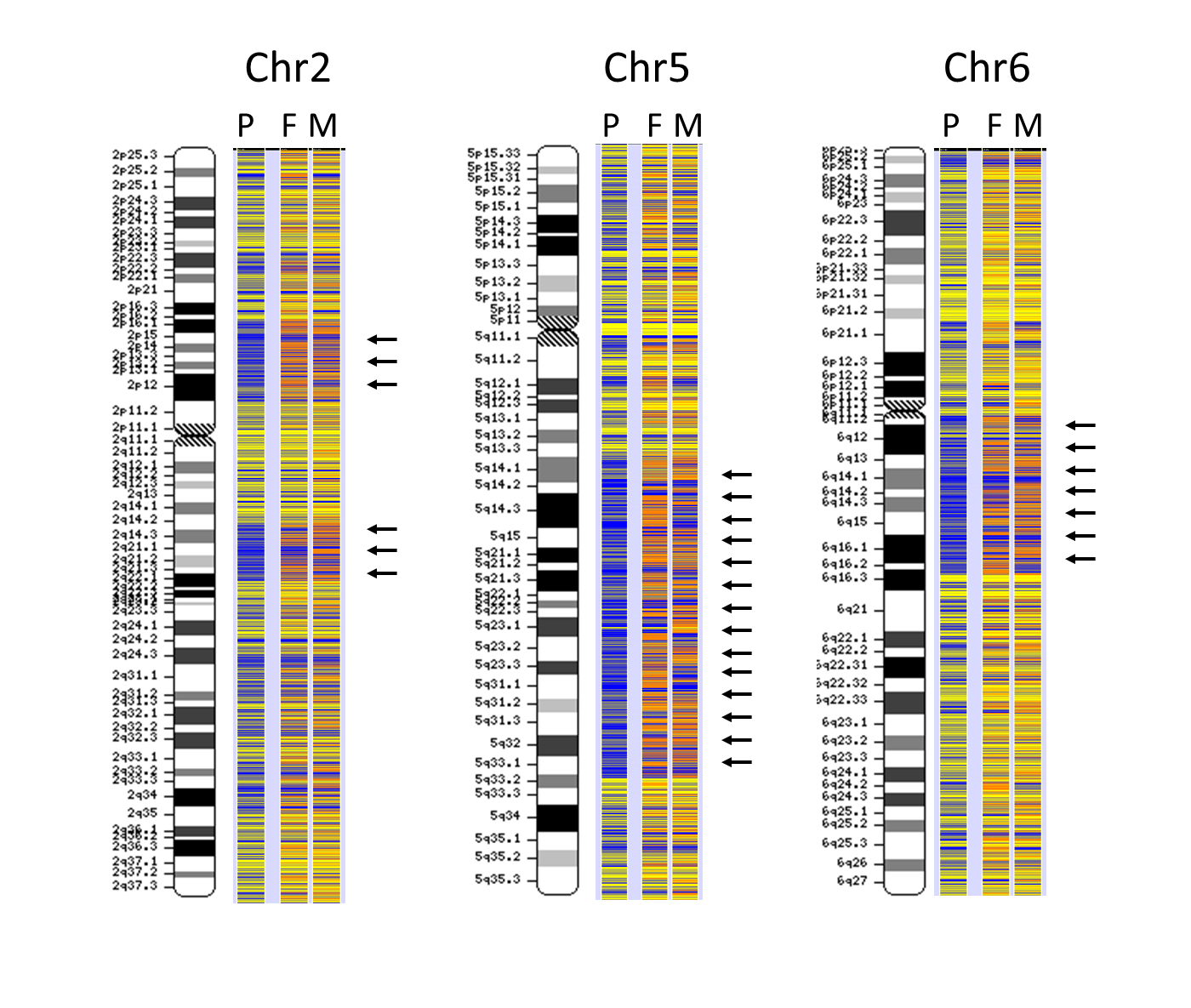

Supplement: Supplementary file 2 — The identified regions of homozygous stretches in chromosome 2, 5 and 6. Description: Blue regions indicate homozygous variants and yellow regions indicate heterozygous variants. Orange regions in parental chromosomes (F, M) indicate heterozygous variants corresponding with homozygous variants of sibling’s genotype (P). (TIFF 898 kb) [file 13023_2017_682_MOESM2_ESM.tif]

**a.**

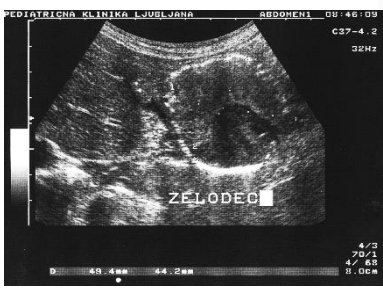

**b.**

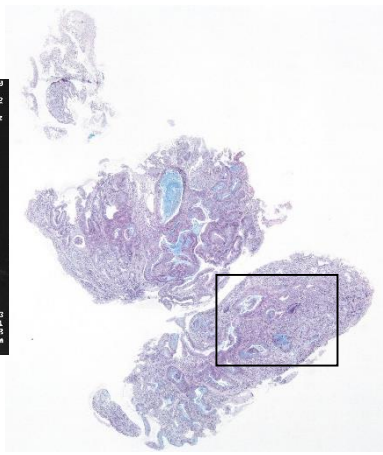

**c.**

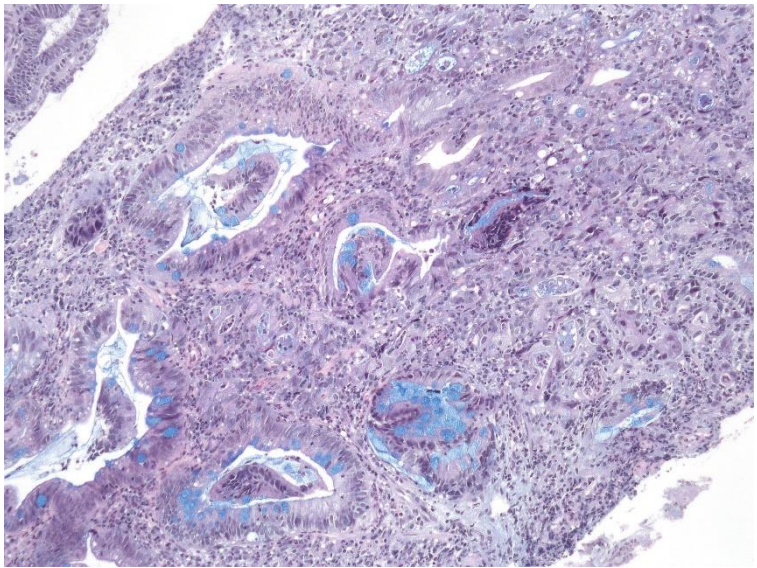

Supplement: Supplementary file 5 — Ultrasound and histopathologic images of the gastric cancer. Description: (a.) Ultrasound image of a 49 × 44 mm large tumor formation in the stomach; (b., c.) histopathologic images of bioptic material from the gastric tumor demonstrating intestinal type gastric carcinoma with lamina propria invasion (Kreyberg trichrom stain, 20X magnification (b.) 100X magnification (c.)). The square in part b. indicates the area of magnification in part c. of the figure. (PDF 223 kb) [file 13023_2017_682_MOESM5_ESM.pdf]
