# Supplementary material for: Multifocal gastric adenocarcinoma in a patient with LRBA deficiency
Source: Orphanet J Rare Dis. 2017 Jul 18;12:131. doi: 10.1186/s13023-017-0682-5 (PMC5516372; doi:10.1186/s13023-017-0682-5)
Supplement: Supplementary file 3 — Autoantibodies evaluated in the patient. (DOCX 12 kb) [file 13023_2017_682_MOESM3_ESM.docx]

**Table S1. Autoantibodies evaluated in the patient.**

| Patient's age (years)  Antibodies | 1.5 | 3 | 12 | 14 | 16 | 19 |
| --- | --- | --- | --- | --- | --- | --- |
| IgG AGA | neg |  |  |  |  | poz |
| IgA AGA | neg |  |  |  |  | poz |
| tTG | ND |  |  |  |  | neg |
| EMA | ND |  |  |  |  | neg |
| Anti-enterocyte Ab |  |  | neg | neg |  |  |
| ANA |  | poz | poz | neg |  | neg |
| antiDNA |  | poz |  | neg |  | neg |
| SMA |  |  |  | neg | neg |  |
| pANCA |  |  |  |  | poz | poz |
| AMA |  |  |  |  |  | neg |
| ASCA |  |  |  |  |  | neg |
| PR3-ANCA |  |  |  |  | neg |  |

Legend: ND – not determined, AGA (IgG, IgA-class antigliadin antibodies), EMA (endomysial antibodies), tTG (tissue transglutaminase antibodies) ANA – anti-nuclear antibodies, SMA ( smooth muscle antibodies), pANCA (perinuclear antineutrophil cytoplasmic antibody), AMA (antimitochondrial antibody , ASCA (anti-Saccharomyces cerevisiae antibodies), PR3-ANCA (anti-neutrophil cytoplasmic antibodies)
