# Supplementary material for: Multifocal gastric adenocarcinoma in a patient with LRBA deficiency
Source: Orphanet J Rare Dis. 2017 Jul 18;12:131. doi: 10.1186/s13023-017-0682-5 (PMC5516372; doi:10.1186/s13023-017-0682-5)
Supplement: Supplementary file 4 — Selected immune system determinants longitudinally evaluated in the patient. (DOCX 15 kb) [file 13023_2017_682_MOESM4_ESM.docx]

**Table S2. Selected immune system determinants longitudinally evaluated in the patient.**

|  | Before gastric cancer | | After gastric cancer | |
| --- | --- | --- | --- | --- |
| Age (years) | 10 | 17 | 26 | 32 |
| *Lymphocyte subsets*  *(absolute number (%) [normal range])* |  |  |  |  |
| CD3+ cells | NA (72%) | 399 (87%)  [ 1100 -1700 cells /mm^3^] | 0.252(71%)  [0.7-1.9 x 10^9^ cells/L] | 0.474 (88%)  [0.7-1.9 x 10^9^ cells/L] |
| CD4+ cells | NA (45%) | 253 (55%)  [700-1100 cells/ mm^3^] | 0.169 (46%)  [0.4-1.3 x 10^9^ cells/L] | 0.317 (59%)  [0.4-1.3 x 10^9^ cells/L] |
| CD8+ cells | NA (35%) | 156 (34%)  [500-900 cells/ mm^3^] | 0.080 (22%)  [0.2-0.7 x 10^9^ cells/L] | 0.130 (24%)  [0.2-0.7 x 10^9^ cells/L] |
| CD19+ cells | NA (10%) | 9 (2%)  [200-400 cells/ mm^3^] | 0.004 (1%)  [0.1-0.4 x 10^9^ cells/L] | 0.004 (1%)  [0.1-0.4 x 10^9^ cells/L] |
| CD4/CD8 | 1.28 | 1.62 | 2.1 | 2.44 |
| CD3HLA-DR cells | NA | 41 cells/ mm^3^ (9%) | NA | 0.455 (96%)  [0.04-0.2 x 10^9^ cells/L] |
| CD25+CD4+ cells | NA | NA | NA | 0.4% [1-5%] |
| CD16+CD56 cells | NA | 64 (14%)  [200-400 cells/ mm^3^] | 0,094(27%)  [0.1-0.4 x 10^9^ cells/L] | 0.062 (12%)  [0.1-0.4 x 10^9^ cells/L] |
| *Mitogen proliferation assay* |  |  |  |  |
| PHA | NA | NA | NA | 14% [27%-48%] |
| CD3, CD28 | NA | NA | NA | 70% [50%-85%] |
| *Immunoglobulins (g/L)* |  |  |  |  |
| IgG (6.9 – 14.0) | 8.61 | 6.1 | 3.73 | NI |
| IgA (0.7 – 4.1) | 0.73 | 0.6 | 2.27 | NI |
| IgM (0.3 – 2.4) | 0.73 | 0.3 | 0.22 | NI |

**Legend:** CD (cluster of differentiation), NA (not available), PHA (phytohemagglutinin), NI (not informative, patient was receiving intravenous immunoglobuline therapy)
